# Retroflex tongue as a non-invasive neurological marker of functional severity in older adults with ischemic stroke: a retrospective observational study

**DOI:** 10.3389/fneur.2025.1632351

**Published:** 2025-08-20

**Authors:** Yung-Sheng Huang, Hen-Hong Chang, John Y. Chiang, Po-Chi Hsu, Lun-Chien Lo

**Affiliations:** ^1^Graduate Institute of Chinese Medicine, China Medical University, Taichung, Taiwan; ^2^Department of Traditional Chinese Medicine, Changhua Christian Hospital, Changhua, Taiwan; ^3^Graduate Institute of Integrated Medicine, China Medical University, Taichung, Taiwan; ^4^Chinese Medicine Research Center, China Medical University, Taichung, Taiwan; ^5^Department of Chinese Medicine, China Medical University Hospital, Taichung, Taiwan; ^6^Department of Healthcare Administration and Medical Informatics, Kaohsiung Medical University, Kaohsiung, Taiwan; ^7^Department of Computer Science and Engineering, National Sun Yat-sen University, Kaohsiung, Taiwan; ^8^School of Chinese Medicine, China Medical University, Taichung, Taiwan

**Keywords:** ischemic stroke, retroflex tongue, tongue deviation, neurological markers, stroke severity

## Abstract

**Background:**

With the growing global burden of ischemic stroke in aging populations, there is increasing interest in simple and non-invasive neurological markers to support early risk stratification and functional prognosis. Retroflex tongue (RT) and tongue deviation (TD) are observable signs of cranial nerve dysfunction; however, their comparative predictive value for stroke severity remains unclear.

**Methods:**

In this retrospective observational study, 308 older adults (mean age: 69.1 years) hospitalized with ischemic stroke were enrolled. Tongue motor function was evaluated using an automatic tongue diagnosis system (ATDS). Deviation angles were measured, and the presence or absence of RT was assessed by trained traditional Chinese medicine physicians. Stroke severity and functional outcomes were evaluated using the National Institutes of Health Stroke Scale (NIHSS), Barthel Index, and length of hospital stay.

**Results:**

Among the 308 patients, 59 (19.2%) exhibited TD and 249 (80.8%) did not. No significant differences were found in baseline characteristics between TD groups, except for deviation angle (TD: 9.72° ± 8.91° vs. non-TD: 6.40° ± 7.84°, *p* = 0.011). Patients without RT had significantly worse clinical outcomes, including longer hospital stays (32.0 vs. 25.9 days, *p* = 0.007), higher NIHSS scores (14.1 vs. 8.9, *p* < 0.001), and lower Barthel Index scores (18.6 vs. 35.0, *p* < 0.001), compared to those with RT. In contrast, TD showed no significant association with these outcomes. Multivariate regression identified non-RT as an independent predictor of stroke severity. ROC analysis supported the discriminative value of RT (AUC = 0.703 for NIHSS ≥ 9; AUC = 0.712 for Barthel ≤ 17), whereas TD showed poor predictive ability (AUC = 0.524 and 0.570, respectively).

**Conclusion:**

Absence of retroflex tongue is more strongly associated with stroke severity and functional impairment than tongue deviation. As a simple, observable motor sign, RT may serve as a practical bedside indicator for early neurological assessment. However, given its preliminary status, further validation in prospective, multi-center studies is warranted before clinical application.

## Introduction

Stroke remains one of the leading causes of mortality and long-term disability worldwide, particularly in aging populations. Current epidemiological data indicate that nearly 30 million people globally have experienced a stroke, with approximately 70% of these cases attributed to ischemic stroke. Stroke contributes to an estimated 58% of the 113 million global disability-adjusted life years (DALYs), underscoring its profound public health burden (1, 2). The absolute number of stroke cases, stroke deaths, and DALYs caused by stroke increased by 70.0, 43.0, and 32.0%, respectively, from 1990 to 2019 (3). Common post-stroke neurological impairments include motor paralysis, dysphagia, aphasia, and cognitive dysfunction, making early identification of functional severity and timely rehabilitation critical to optimizing outcomes (4). Among them, dysphagia occurs in 29 to 67% of post-stroke patients and leads to serious complications, such as aspiration, dehydration, malnutrition. Moreover, the movement of the tongue plays an important contributor to the oral-stage swallowing (5, 6).

Clinically, stroke is not only associated with limb motor dysfunction but also affects tongue mobility due to damage to the hypoglossal nerve. Tongue deviation (TD), characterized by a deviation of the tongue toward the side of the lesion, is a common manifestation of such neurological impairment (7, 8). Prior studies have reported that TD is associated with dysphagia in 43% of stroke patients and dysarthria in up to 90%, suggesting that it may serve as an early clinical sign of brainstem involvement (9). Moreover, a tongue deviation angle exceeding 3.2 degrees has been proposed as a potential indicator of stroke risk (10).

**Figure 1:**
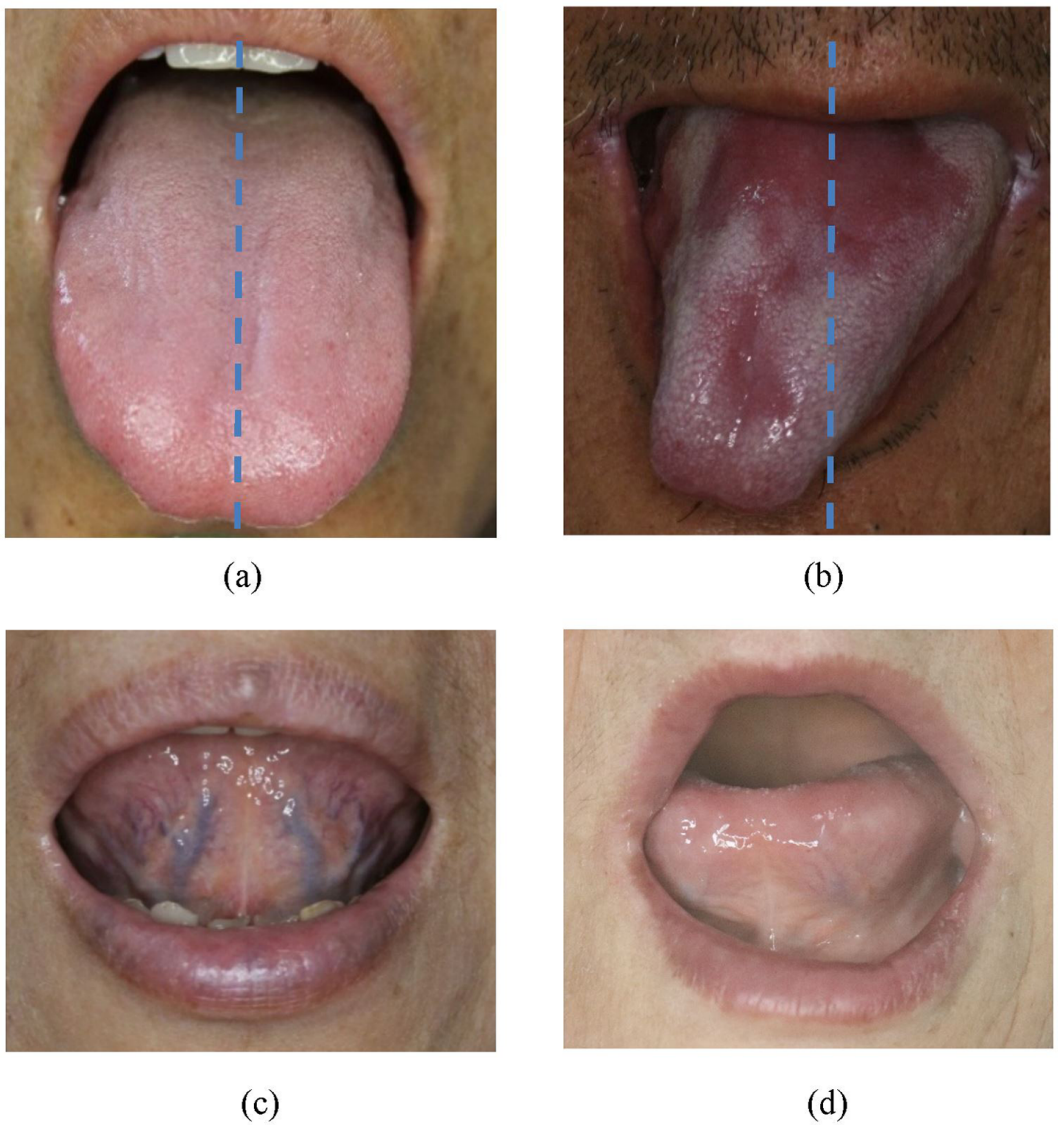
**(a)** Non-tongue deviation (normal posture without tongue deviation). **(b)** Tongue deviation (the tip of tongue severely slanted to one side, away from midline of upper lip). **(c)** Retroflex tongue (a tongue can freely curl up and expose the sublingual veins for observation). **(d)** Non-retroflex tongue (tongue no curling up of tongue at all).

In our previous study, we demonstrated that retroflex tongue (RT), the ability to curl the tongue tip upward, was significantly associated with stroke severity and functional prognosis. Patients lacking RT capability (non-RT) showed longer hospital stays (32.0 ± 21.5 vs. 25.9 ± 14.4 days, *p* value: 0.007) and significantly worse NIHSS (14.1 ± 7.8 vs. 8.9 ± 5.2, *p* value < 0.001) and Barthel Index scores (18.6 ± 20.7 and 35.0 ± 24.2, *p* value < 0.001). Moreover, the non-RT patients account for 60.2 and 75.6% for Barthel Index ≤ 17 and NIHSS ≥ 9 according to the ROC curve of retroflex tongue (11). The schematic diagram of RT is as Figure 1. Other study indicates that forward and backward movement of the tongue (such as tongue protrusion and retraction) is a more important factor in oral swallowing than lateral movement of the tongue, according to anterior hard palate-to-tongue pressure and posterior hard palate-to-tongue pressure during swallowing (12). However, no study has yet evaluated the combined prognostic utility of TD and RT within the same patient cohort.

From a translational and geriatric neurology perspective, simple, non-invasive clinical signs such as TD and RT may offer practical value in early risk stratification, especially in primary care or resource-limited settings. These signs may support timely triage, enhance functional assessment, and inform rehabilitation planning for older adults with ischemic stroke. This study aims to evaluate TD and RT concurrently and assess their independent and combined associations with stroke severity, thereby identifying novel bedside markers for functional prognosis in aging stroke populations.

## Materials and methods

### Study design and population

This was a retrospective, cross-sectional study. The patients with stroke admitted to Changhua Christian Hospital (CCH) and consulted TCM treatment from August 1st, 2010, to July 31st, 2013, were recruited. The tongue database was jointly collected by the Department of TCM and Stroke Center in CCH. A total of 317 patients were included, 2 were excluded due to unclear photographs, and 7 were excluded due to incomplete data collection, leaving 308 complete cases for analysis. Patient information is coded, using numbers or English letters to replace the information such as provider’s name, ID number, medical record number, which can be used to identify personal information. Participants were introduced to the purpose, procedures, potential risks, and benefits of the study first, following which they provided informed consent. The study was approved by Institutional Review Board of CCH, Taiwan (IRB No.150110).

*Inclusion Criteria:* Participants meeting the following criteria would be included (1) Participants diagnosed as ischemic stroke (ICD-9:433∼438) by neurologist and approved by head CT or MRI examination. (2) The period from ischemic stroke diagnosis to tongue examination less than 6 months. (3) Age 20 years old or older. (4) Complete tongue diagnosis data.

*Exclusion Criteria*: Participants meeting one or more of the following criteria would be excluded (1) Hemorrhagic stroke (ICD-9: 430∼432) (2) History of ischemic heart disease, including acute myocardial infarction (ICD-9: 410) and other ischemic heart diseases (ICD-9: 411–414) (3) Unstable vital sign or unconsciousness (4) Unable to protrude tongue or with insufficient length protruded to allow capturing of tongue image (5) Cognitive impairment or unable to communicate.

### ATDS and tongue motor status

The tongue images of the participants were collected by the validated Automatic Tongue Diagnosis System (ATDS). The ATDS was developed by our team to capture tongue images and automatically extract features consistently to assist the diagnosis of TCM practitioners. There are some peer-reviewed researches show ATDS has been validated for high consistency (13–15). Tongue motor status included both tongue deviation and retroflex tongue. Non-retroflex tongue signifies difficulty in curling up the tongue tip to expose the sublingual veins for observation. Tongue deviation signifies that the tongue turns away from the midline when extended or protruded, and will deviate toward the side of the lesion. The diagnosis of tongue deviation was recognized by five well-trained TCM physicians, and the angle of tongue deviation between the middle line of tongue and lip was identified by ATDS.

### Data collection

Information including demography, body mass index (BMI), hemoglobin A1c (HbA1c), cholesterol and triglyceride (TG) were gathered for each subject. Data collected included ICD-9 code, the time period from ischemic stroke diagnosis to tongue examination, the admission date and discharge date, NIHSS score and Barthel Index score on admission, stroke-related diseases (diabetes, hypertension, hyperlipidemia, etc.). Tongue images were collected for each subject to further derive the relevant tongue features of every participant. All personal details and photographs of subjects recruited were encrypted to ensure confidentiality.

### Statistical analysis

All the analyses were conducted using SPSS 22.0 statistical software. Chi-square test or Fischer’s exact test was utilized to analyze the nominal variables, while Independent-Samples 𝑡-test was employed for the continuous ones. Furthermore, One-way ANOVA followed by Bonferroni *post hoc* correction was used to control for multiple comparisons when evaluating differences between subgroups based on retroflex tongue and tongue deviation status. The receiver operating characteristic (ROC) curve was assessed to investigate the relationship between RT and TD with NIHSS and Barthel Index. 𝑝 value < 0.05 was considered statistically significant.

## Results

The clinical characteristics of patients with ischemic stroke are listed in Table 1. Tongue deviation served as the discriminating criterion to segment the stroke cases into two clusters: the with and without tongue deviation groups. A total of 59 patients (19.2%) with tongue deviation and 249 patients (80.8%) without tongue deviation were enrolled in this study. No significant difference was observed in criteria such as age, BMI, gender, history of stroke-related diseases (diabetes, hypertension, and hyperlipidemia), time of first incidence of stroke, Time from stroke diagnosed to tongue examination, and laboratory data (HbA1c, T-cholesterol, Triglyceride, and HDL) between those with tongue deviation and those without tongue deviation. There was significant difference with the angle of tongue deviation by ATDS, with 6.40 ± 7.84 vs. 9.72 ± 8.91 (*p* value = 0.011).

**Table 1:** The basic data of ischemic stroke patients between tongue deviation groups.

Variable	Tongue deviation		*p*-value
	With (*N* = 59)	Without (*N* = 249)	
Gender			0.831
Male, *N* (%)	21 (35.6)	85 (34.1)	
Female, *N* (%)	38 (64.4)	164 (65.9)	
Age, Mean (SD)	70.0 (11.2)	68.8 (12.2)	0.484
BMI, Mean (SD)	24.8 (4.2)	24.2 (4.0)	0.364
Comorbidity			
DM, *N* (%)	23 (39.0)	103 (41.1)	0.738
HTN, *N* (%)	48 (81.4)	203 (81.5)	0.976
Hyperlipidemia, *N* (%)	34 (57.6)	126 (50.6)	0.332
First-time stroke, *N* (%)	46 (78.0)	195 (78.6)	0.211
Non-retroflex tongue, *N* (%)	24 (40.7)	99 (39.8)	0.857
HbA1C (%), Mean (SD)	6.8 (2.0)	7.0 (2.1)	0.571
Total cholesterol (mg/dL), Mean (SD)	178.3 (45.6)	181.1 (43.4)	0.700
Triglyceride (mg/dL), Mean (SD)	128.4 (119.4)	120.9 (75.7)	0.590
HDL (mg/dL), Mean (SD)	55.0 (49.4)	40.2 (16.3)	0.592
Time from stroke diagnosed to tongue examination (days), Mean (SD)	22.22 (19.37)	23.17 (22.64)	0.769
Angle of deviation (degree), Mean (SD)	9.72 (8.91)	6.40 (7.84)	0.011^*^

Student’s t test and Chi-Square test or Fisher’s Exact test. ^*^p < 0.05 has significant statistical differences.

Differences of severity assessment of stroke patients with ischemic stroke between retroflex tongue and tongue deviation are shown in Table 2. The ischemic stroke patients who cannot retroflex their tongues had longer length of stay than their RT counterparts, 32.0 ± 21.5 days vs. 25.9 ± 14.4 days (*p* value = 0.007). The average NIHSS score on admission was higher in the non-RT group than that of the RT group: 14.1 ± 7.8 vs. 8.9 ± 5.2 (*p* value < 0.001). The Barthel Index on admission was also lower in the non-RT group, with 18.6 ± 20.7 vs. 35.0 ± 24.2 (*p* value < 0.001) than in the RT group. However, no significant difference was observed between the tongue deviation and the without tongue deviation group in admission days, NIHSS score, and Barthel Index.

**Table 2:** The severity assessment of ischemic stroke patients between retroflex tongue and tongue deviation groups.

Variable	Retroflex tongue		*p*-value
	With (*N* = 185)	Without (*N* = 123)	
Admission day (days), Mean (SD)	25.9 (14.4)	32.0 (21.5)	0.007^*^
NIHSS score ¡ -Mean (SD)	8.9 (5.2)	14.1 (7.8)	<0.001^*^
Barthel Index ¶-Mean (SD)	35.0 (24.2)	18.6 (20.7)	<0.001^*^

Variable	Tongue deviation		*p*-value
	With (*N* = 59)	Without (*N* = 249)	
Admission day (days), Mean (SD)	27.5 (10.8)	28.5 (19.0)	0.709
NIHSS score ¡ -Mean (SD)	10.9 (6.0)	11.0 (7.0)	0.985
Barthel Index ¶-Mean (SD)	31.6 (21.5)	27.7 (24.7)	0.269

Student’s t test and Chi-Square test or Fisher’s Exact test. ^*^p < 0.05 has significant statistical differences. ¡National Institutes of Health Stroke Scale (NIHSS) ranges from 0 to 42 score, the higher the score means higher neuro-damage. The NIHSS has a total of 15 items. ¶Barthel Index is a common life function scale, ranging from 0 to 100 score. The lower the score, the higher the life-dependency.

The comparisons among admission days, NIHSS score, and Barthel Index between the retroflex tongue and tongue deviation groups are shown in Table 3. Additionally, we divided patients into four groups through tongue retroflex and tongue deviation or not. Consequently, there were significant increases in admission days, NIHSS score and decreases in Barthel Index in the non-RT group, with either tongue deviation or not. Non-RT plays a dominant role in the progression of ischemic stroke. Furthermore, according to the analysis result of the Bonferroni *post hoc* test, the NIHSS score means of the non-retroflex tongue groups were higher than the means for retroflex tongue patients. Under the admission days variable, only non-deviation tongue with or without retroflex tongue was verified as statistically significant. Additionally, the Barthel Index signified statistical significance between non-retroflex tongue without deviation group and retroflex tongue with or without deviation.

**Table 3:** The result of Bonferroni multiple comparisons between retroflex tongue and tongue deviation groups.

Variable	Retroflex tongue		Non-retroflex tongue		*p*-value
	Non*-TD^a^ (*N* = 150)	TD^b^ (*N* = 35)	Non-TD^c^ (*N* = 99)	TD^d^ (*N* = 24)	
Admission day (day), Mean (SD)	25.9 (15.7)	25.7 (6.0)	32.3 (22.8)	30.3 (15.4)	0.032^*^
NIHSS score Mean (SD)	8.9 (5.3)	9.1 (4.8)	14.2 (8.1)	13.7 (6.5)	<0.001^*^
Barthel Index Mean (SD)	34.4 (24.7)	37.7 (21.8)	17.6 (21.2)	22.7 (18.1)	<0.001^*^

*p*-value by One-Way ANOVA follow with Bonferroni multiple comparisons at type I error of 0.05 level. DT, tongue deviation; Post Hoc tests: Admission days, a < c. NIHSS score, a, b < c, d. Barthel Index, a, b > c. *p < 0.05.

The multiple linear regression between retroflex tongue and tongue deviation on NIHSS of patients with ischemic stroke is shown in Table 4. In addition, we conducted One-Way ANOVA followed by multiple linear regression analysis to examine which variables: age, gender, BMI, retroflex tongue and tongue deviation, determine the NIHSS of ischemic stroke patients. We found that the variables of patients’ age, gender, BMI and retroflex tongue bore no significant difference on NIHSS. Only the non-retroflex tongue groups showed significant statistical differences (*p*-value = 0.001). Non-retroflex tongue without deviation tongue and non-retroflex tongue with tongue deviation showed statistical significance. As identified in the NIHSS score, non-retroflex tongue has a higher correlation than tongue deviation.

**Table 4:** The results of multiple linear regression between retroflex tongue and tongue deviation on NIHSS of ischemic stroke patients.

Predictor		Estimate	SE	95% C.I.			*p*-value
Age		0.003	0.002	−0.001	-	0.008	0.173
Gender	Male	−0.066	0.058	−0.179	-	0.048	0.256
	Female	0.000					
BMI		−0.003	0.007	−0.017	-	0.010	0.628
Retroflex tongue (*N* = 185)	Without TD (*N* = 150)	0.000					
	With TD (*N* = 35)	−0.047	0.088	−0.219	-	0.125	0.594
Non-retroflex tongue (*N* = 123)	Without TD (*N* = 99)	0.300	0.061	0.181	-	0.420	<0.001^*^
	With TD (*N* = 24)	0.337	0.103	0.135	-	0.539	0.001^*^

*p*-value by One-Way ANOVA follow with multiple linear regression, ^*^p < 0.05, Bonferroni-adjusted. Multiple linear regression followed by Bonferroni correction. TD, tongue deviation.

**Figure 2:**
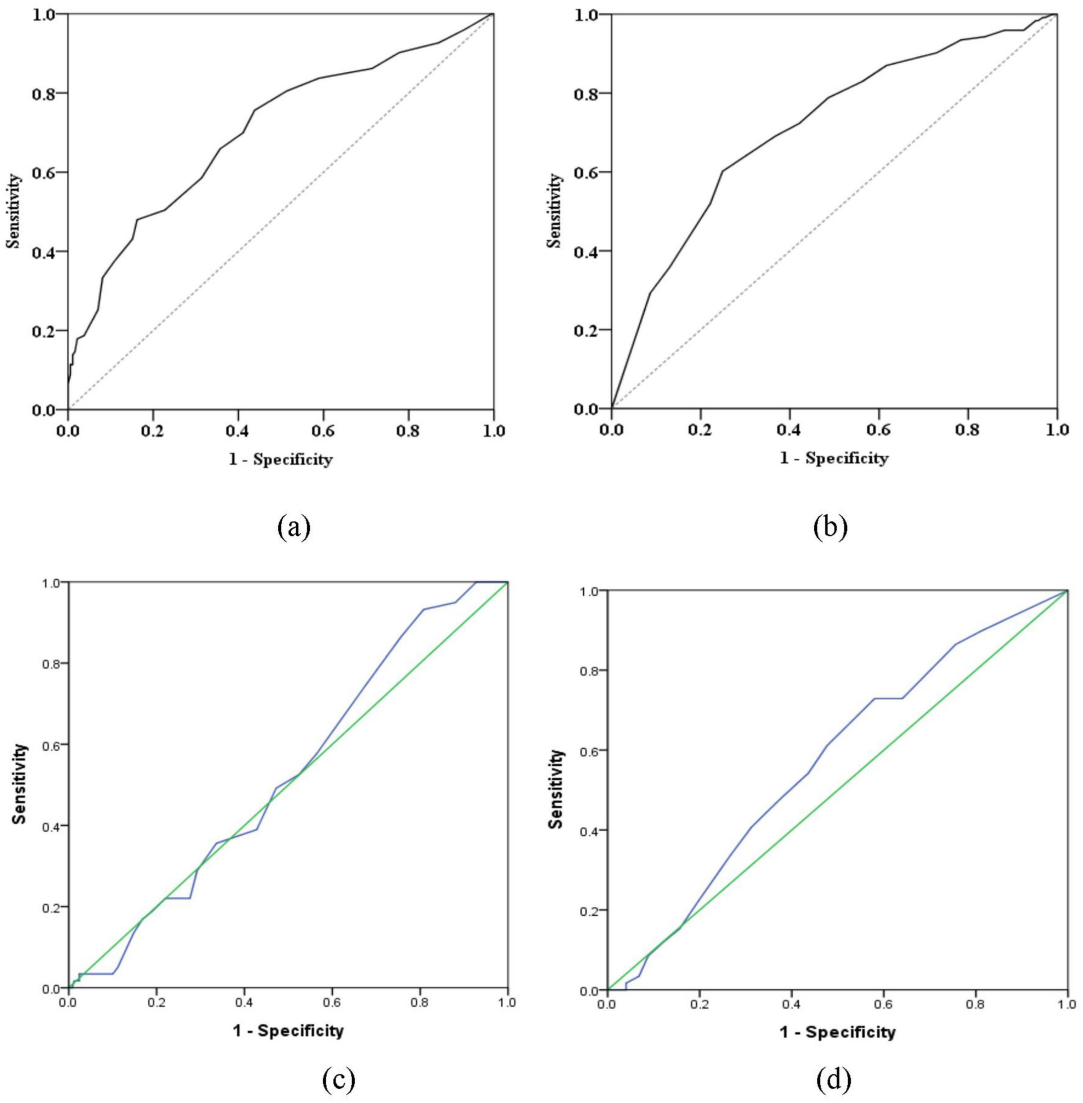
The receiver operating characteristic curve (ROC curve) between retroflex tongue and tongue deviation. **(a)** The ROC curve between RT and NIHSS score values. **(b)** The ROC curve between RT and Barthel Index. **(c)** The ROC curve between TD and NIHSS score values. **(d)** The ROC curve between TD and Barthel Index. These figures are partly available in the previous publication (11) such as Figures 2a,b (licensed under CC BY 4.0).

According to the receiver operating characteristic curve (ROC curve), we assessed the relationship between retroflex tongue and tongue deviation with NIHSS and Barthel Index in ischemic stroke patients. Youden’s index (sensitivity + specificity − 1) for retroflex tongue showed the highest values of 0.318 and 0.353 in NIHSS scores ≧ 9 and Barthel ≦ 17, respectively. The area under the curve was 0.703 and 0.712, with *p*-values < 0.001 and < 0.001. However, Youden’s index for tongue deviation showed lower values of 0.124 and 0.034 in NIHSS scores ≧ 5 and Barthel ≦ 73. The area under the curve was 0.524 and 0.570, with p-values = 0.563 and = 0.096. The data was presented in Table 5, and the ROC curves were presented in Figure 2.

**Table 5:** The ROC curve of retroflex tongue and tongue deviation between NIHSS and Barthel Index of ischemic stroke patients.

Criterion values and coordinates of ROC curve					Area under the ROC curve					
Value	Sensitivity	Specificity	+LR	−LR	Area	SE	95% C.I.			*p*-value
The ROC curve of retroflex tongue and NIHSS and Barthel index of ischemic stroke patients										
NIHSS ≥ 9	0.756	0.562	1.727	0.434	0.703	0.031	0.643	-	0.764	<0.001
Barthel Index ≤ 17	0.602	0.751	2.420	0.530	0.712	0.030	0.653	-	0.771	<0.001
The ROC curve of tongue deviation and NIHSS and Barthel Index of ischemic stroke patients										
NIHSS ≥ 5	0.932	0.192	1.530	0.353	0.524	0.038	0.449	-	0.599	0.563
Barthel Index ≤ 73	0.966	0.068	1.037	0.499	0.570	0.039	0.493	-	0.647	0.096

Sensitivity, true positive rate; Specificity, true negative rate. LR+, positive likelihood ratio; LR−, negative likelihood ratio. LR+ = Sensitivity/(1 − Specificity); LR− = (1 − Sensitivity)/Specificity. Area, Area under the curve.

## Discussion

This study presents a comprehensive evaluation of two clinically observable tongue motor signs, TD and RT in patients with ischemic stroke, using objective metrics obtained via the ATDS. Our findings indicate that the absence of retroflex tongue (non-RT) is significantly associated with greater stroke severity and poorer functional outcomes, as measured by NIHSS score, Barthel Index, and length of hospital stay. In contrast, TD alone did not demonstrate a statistically significant correlation with these clinical indicators. These findings suggest that retroflexibility may serve as a more sensitive and clinically relevant marker of neurological dysfunction in aging stroke populations.

Tongue diagnosis has long been recognized as a non-invasive diagnostic method in Traditional Chinese Medicine (TCM), offering insights into internal physiological states such as qi and blood flow, as well as yin-yang balance (16, 17). Recent advances in computerized tongue analysis have expanded its application to a range of diseases, including rheumatoid arthritis, breast cancer, diabetes, and metabolic syndrome (18–23). However, its integration into cerebrovascular or neurodegenerative disease assessment remains limited. Prior studies on TD has primarily focused on its presence as a clinical symptom and its relationship with hypoglossal nerve dysfunction, but the use of objective deviation angles and its comparison with other tongue motor signs have been limited.

This study is the first to directly compare TD and RT within the same patient cohort. Our data showed that while 19.2% of stroke patients presented with TD, the deviation angle measured via ATDS was significantly higher in the TD group (9.72° ± 8.91°) than in the non-TD group (6.40° ± 7.84°, *p* = 0.011), which corroborates prior observations that TD is a recognizable feature of lower cranial nerve dysfunction. However, when TD and non-RT were compared in relation to functional outcomes, only non-RT demonstrated statistically significant associations. One-way ANOVA revealed that NIHSS scores were significantly elevated in patients with non-RT, whereas no such difference was observed in TD subgroups. This finding suggests that retroflexibility may reflect a broader spectrum of neuroanatomical involvement, beyond focal hypoglossal deficits. Although the TD angle was measured quantitatively via ATDS, it was modeled as a categorical variable in our regression analysis, consistent with clinical convention and diagnostic thresholds reported in the literature. We acknowledged that this may reduce analytical sensitivity, and future studies should consider modeling TD as a continuous variable to better capture its relationship with neurological impairment.

One possible explanation for this discrepancy lies in the underlying neural mechanisms. TD typically results from unilateral damage to the hypoglossal nerve, which affects tongue protrusion and causes deviation toward the lesioned side (24–26). In contrast, the ability to retroflex the tongue likely involves more complex neural control, potentially engaging both cortical, subcortical, and brainstem pathways. We hypothesize that non-retroflexibility may signify damage extending beyond the hypoglossal nucleus, implicating cortical or corticobulbar tract involvement. Given that age-related cerebrovascular and neurodegenerative changes frequently impact distributed motor control networks, retroflexibility could serve as a surrogate marker of such diffuse neurological impairment.

Our findings are consistent with prior work, which demonstrated the prognostic value of RT in stroke patients (11). This study builds upon that foundation by introducing a comparative analysis with TD, thereby advancing the understanding of differential tongue motor signs as potential bedside prognostic tools. The use of ATDS allows for objective quantification, enhancing reproducibility and minimizing examiner bias.

Several limitations should be acknowledged. First, the single-center retrospective design may introduce inherent risks of selection bias and information bias. This may limit the generalizability of our findings to broader ischemic stroke populations or to other clinical settings. Second, we were unable to provide detailed neuroimaging findings (e.g., lesion location or infarct volume) or precise timing of tongue assessments (e.g., distinguishing between acute, subacute, and chronic phases). This limitation precluded stratified analyses by stroke phase and may have influenced the interpretation of tongue motor function changes over time. Future studies should consider stratifying patients by stroke phase and incorporating longitudinal follow-up to better capture the temporal dynamics of tongue motor recovery. Third, the assessment of RT was based solely on physicians’ visual judgment, without the application of objective or quantifiable ATDS criteria. We recommend the integration of ATDS for RT evaluation in future work to improve standardization. Forth, inter- and intra-rater reliability (e.g., Cohen’s kappa) for RT and TD classification was not formally assessed in this study, although a standardized training protocol was applied. We recognize this as a methodological limitation and aim to incorporate these analyses in future validation studies. Finally, information regarding acute interventions such as thrombolysis or thrombectomy was not available, which may have affected clinical outcomes and tongue motor function assessments.

Given these limitations, RT should be regarded as a preliminary clinical finding. While our data demonstrate a statistically significant association between RT absence and stroke severity, this finding should be interpreted cautiously. RT is not currently validated as an independent diagnostic or prognostic biomarker. Its potential clinical utility requires confirmation through well-designed prospective, multi-center studies with standardized assessment protocols and objective measurements.

Besides, we propose that future studies integrate artificial intelligence (AI)-based approaches, such as automated video recognition and machine learning algorithms, to improve the accuracy, consistency, and scalability of RT assessment. For example, deep learning models trained on video data could capture dynamic tongue movements and provide objective classification based on standardized features.

## Conclusion

The absence of retroflex tongue (non-RT) appears to be more strongly associated with stroke severity and functional impairment than tongue deviation. This observable motor sign, assessable through brief bedside inspection, may offer practical utility for early neurological risk stratification, particularly in primary care or low-resource settings. However, the interpretation of RT status should remain cautious. At present, RT represents a preliminary clinical observation rather than a validated diagnostic or prognostic indicator. Additional prospective studies with larger, diverse populations are required to establish its clinical relevance. Our findings suggest that retroflex tongue has potential as a low-cost, scalable neuromotor marker for assessing functional outcomes in older adults with ischemic stroke, but further validation is essential before it can be incorporated into routine stroke evaluation frameworks.

## Data Availability

The data that support the findings of this study are partly available in the previous publication at doi: 10.1155/2017/3195749, such as Figures 2a,b. Access to the figures may be subject to the Creative Commons Attribution License, which permits unrestricted use, distribution, and reproduction in any medium, provided the original work is properly cited. The data that support the findings of this study are available from the corresponding author, Lun-Chien Lo, upon reasonable request.
